# Viruses of a key coral symbiont exhibit temperature-driven productivity across a reefscape

**DOI:** 10.1038/s43705-023-00227-7

**Published:** 2023-04-03

**Authors:** Lauren I. Howe-Kerr, Carsten G. B. Grupstra, Kristen M. Rabbitt, Dennis Conetta, Samantha R. Coy, J. Grace Klinges, Rebecca L. Maher, Kaitlin M. McConnell, Sonora S. Meiling, Adriana Messyasz, Emily R. Schmeltzer, Sarah Seabrook, Jordan A. Sims, Alex J. Veglia, Andrew R. Thurber, Rebecca L. Vega Thurber, Adrienne M. S. Correa

**Affiliations:** 1 Department of BioSciences, Rice University, Houston, TX USA; 2 Department of Biology, Boston University, Boston, MA USA; 3 Department of Marine and Environmental Sciences, Northeastern University, Boston, MA USA; 4 Department of Oceanography, Texas A & M University, College Station, TX USA; 5 Mote Marine Laboratory, Elizabeth Moore International Center for Coral Reef Research & Restoration, Summerland Key, FL USA; 6 Institute of Ecology and Evolution, University of Oregon, Eugene, OR USA; 7 Oregon State University, Corvallis, OR USA; 8 University of the Virgin Islands, St. Thomas, US Virgin Islands USA; 9 Rutgers School of Environmental and Biological Sciences, New Brunswick, NJ USA; 10 National Institute of Water and Atmospheric Research, Wellington, New Zealand; 11 Environmental Science and Policy, George Mason University, Fairfax, VA USA

**Keywords:** Microbial ecology, Microbial ecology

## Abstract

Viruses can affect coral health by infecting their symbiotic dinoflagellate partners (Symbiodiniaceae). Yet, viral dynamics in coral colonies exposed to environmental stress have not been studied at the reef scale, particularly within individual viral lineages. We sequenced the viral major capsid protein (*mcp*) gene of positive-sense single-stranded RNA viruses known to infect symbiotic dinoflagellates (‘dinoRNAVs’) to analyze their dynamics in the reef-building coral, *Porites lobata*. We repeatedly sampled 54 colonies harboring *Cladocopium* C15 dinoflagellates, across three environmentally distinct reef zones (fringing reef, back reef, and forereef) around the island of Moorea, French Polynesia over a 3-year period and spanning a reef-wide thermal stress event. By the end of the sampling period, 28% (5/18) of corals in the fringing reef experienced partial mortality versus 78% (14/18) of corals in the forereef. Over 90% (50/54) of colonies had detectable dinoRNAV infections. Reef zone influenced the composition and richness of viral *mcp* amino acid types (‘aminotypes’), with the fringing reef containing the highest aminotype richness. The reef-wide thermal stress event significantly increased aminotype dispersion, and this pattern was strongest in the colonies that experienced partial mortality. These findings demonstrate that dinoRNAV infections respond to environmental fluctuations experienced in situ on reefs. Further, viral productivity will likely increase as ocean temperatures continue to rise, potentially impacting the foundational symbiosis underpinning coral reef ecosystems.

## Introduction

Viruses in natural environments can employ infection strategies along a continuum from lytic to persistent [1]; although some lyse and kill their hosts, others maintain high infection prevalence in a host population without severely damaging infected individuals (e.g., [2–5]). Conditions associated with human activity and climate change, including temperature [3, 6, 7] nutrient pollution [8], and oxygen depletion [9], can drive shifts in the infection strategies that viruses use, with implications for the productivity of these infections. Despite the growing recognition that natural systems contain prevalent viruses with varied infection modes, the drivers of productivity in coral-associated viruses have not been examined at the reef scale.

Coral reefs, climate-threatened generators of marine ecosystem services, are teeming with diverse microbial consortia, including viruses (e.g., [10, 11]). Viral abundance in corals has been associated with elevated temperatures (e.g., [12–14]) and exposure to additional stressors, including excess nutrients [12] and ultraviolet radiation [15]. Observations of diverse viruses under different conditions have inspired hypotheses about their likely roles, ranging from antagonistic agents driving disease [16–19] to beneficial contributors to coral stress tolerance [20–22]. Recently, a metagenomic comparison of viromes from conspecific healthy and bleached corals suggested that some eukaryotic viruses may directly contribute to bleaching signs, whereas certain bacteriophages may be associated with healthy colonies [23]. These patterns reveal what may be a general pattern associated with coral microbial dysbiosis, broadly defined as a deviation from the microbial consortia of healthy holobionts. However, the dynamics and impacts of specific lineages of viruses have yet to be quantified from reef environments.

The inability to track individual reef-associated viral lineages has constrained progress in characterizing viral prevalence and dynamics in coral holobionts. For coral-associated bacteria and Symbiodiniaceae (algal endosymbionts), microbial dysbiosis has frequently been associated with increases or decreases in richness and increased dispersion [24–28] using community analysis approaches. While amplicon sequencing of Symbiodiniaceae and bacteria communities have been widely applied to understand their impacts on coral colony health, viruses lack a common phylogenetically conserved gene like the 16S of bacteria, limiting our ability to characterize viral dynamics in a comparable manner. However, recent developments have enabled the high-throughput characterization of one group of viruses, Symbiodiniaceae-infecting positive-sense, single-stranded RNA viruses (or ‘dinoRNAVs’). Grupstra et al. [29] produced the first temporal characterization of this viral lineage, using nested degenerate primers that amplify the Symbiodiniaceae dinoRNAV major capsid protein (*mcp*, [30]) and translating amplicon sequence variants into unique amino acid sequences. Here, we apply this approach at the reef scale to track the spatial and temporal dynamics of a viral lineage for the first time, spanning a significant thermal stress event, to test for evidence of dysbiosis in their associated coral colonies.

We tracked dinoRNAV *mcp* diversity in the Symbiodiniaceae assemblages of 54 individual colonies of the thermally resistant coral holobiont, *Porites lobata*, from three reef zones (fringing, back and forereef) in Moorea, French Polynesia over a > 3-year period that spanned an extreme thermal stress event in March of 2019. Coral reef zones in Moorea are characterized by distinct environmental conditions [31]. For example, the shallow fringing and back reef zones are characterized by higher temperatures [32] and nutrient concentrations [33] than the deeper and further offshore forereef zone. *P. lobata* typically associates with *Cladocopium* C15 Symbiodiniaceae [34, 35], making it an ideal candidate to investigate dinoRNAV dynamics within a single Symbiodiniaceae species in a thermally robust coral system [36]. By integrating *mcp* amino acid diversity into the context of colony- and reef-scale parameters, we (1) quantify colony-level infection prevalence and persistence over time, (2) identify putative triggers of viral productivity and dysbiosis based on differences in abiotic factors (i.e., thermal stress, reef zone, etc.), and (3) integrate colony health and viral infection data to generate hypotheses regarding the impacts of viral infection on coral holobionts.

## Methods

### Experimental design and sample processing

**Fig. 1:**
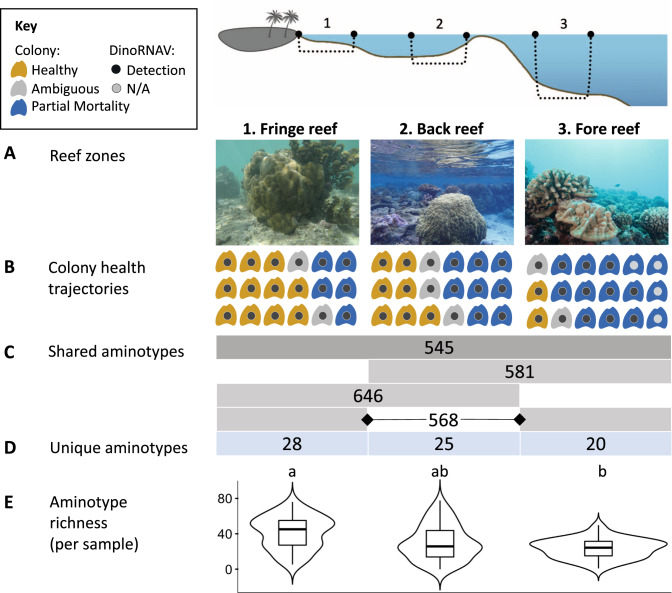
Sampling overview and dinoRNAV detection summary from *Porites lobata* colonies on the reefs of Moorea, French Polynesia (South Pacific). **A** Schematic cross section of sampled reef zones and representative images of focal *Porites lobata* coral colonies in each reef environment. Eighteen colonies per reef zone were tagged and sampled four times from August 2018 to October 2020. **B** Health trajectories of colonies between August 2018 and October 2020 based on image analysis. Orange icons represent colonies that remained visually healthy throughout the sampling duration, whereas blue icons indicate colonies that experienced partial mortality. Gray icons indicate colonies for which health trajectory was ambiguous based on the images available. Dark gray center dots indicate dinoRNAV detection. Light gray “N/A” dots indicates that a colony that was not sequenced due to poor RNA quality. **C** Number of unique dinoRNAV major capsid protein amino acid sequences (‘aminotypes’) detected in samples from all three reef zones (darker gray) or in two of the three zones (lighter gray). **D** Number of aminotypes detected exclusively in colonies within a given reef zone. **E** Boxplot and width distribution of aminotype richness per colony. Letters indicate significant differences in dinoRNAV aminotype richness based on a pairwise Wilcoxon test with Bonferroni correction (fringe vs. back, *p* = 0.10; fringe vs. fore, *p* < 0.01; back vs. fore, *p* = 0.99).

**Fig. 2:**
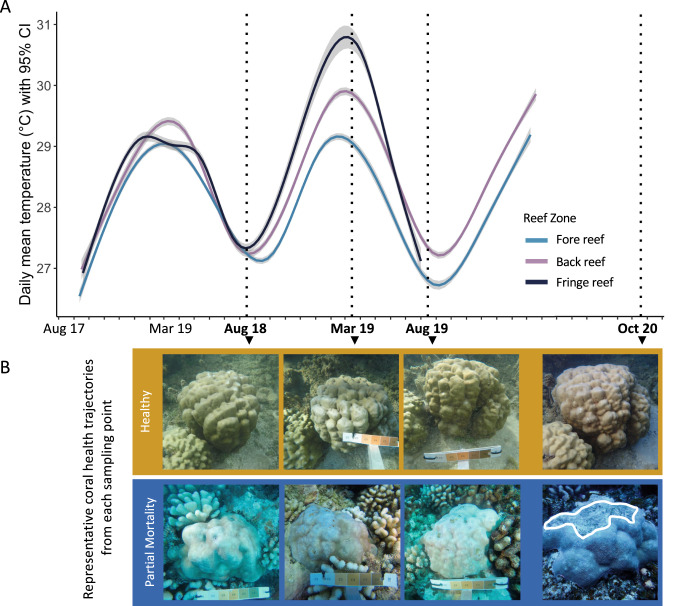
Temperature fluctuated and the health of some *Porites lobata* colonies changed over time on coral reefs of Moorea, French Polynesia (South Pacific). **A** Daily mean temperatures with 95% confidence interval over the sampling duration (temperature data collection ended prior to October 2020). **B** Representative coral health trajectories, depicting one colony that remained healthy at each sampling point over the study duration (orange box) and one colony that experienced partial mortality by October 2020 following bleaching in August 2019 (blue box, partial mortality outlined in white).

Fifty-four colonies of *Porites lobata* were tagged on the north shore of Moorea, French Polynesia, spanning nine sites that encompassed three reef zones (fringing, back and forereef; *n* = 6 colonies/site, *n* = 3 sites/reef zone, Fig. 1A, Supplementary Fig. 1A). Each tagged colony was sampled in August 2018 (dry season), March 2019 (wet season), August 2019 (dry), and October 2020 (dry). Samples could not be collected in March 2020 due to the COVID-19 pandemic. Water temperatures were measured every two hours year-round at each site for the duration of the study using a HOBO® temperature logger. Temperatures are presented as averages (with 95% CI) by reef type (Fig. 2A), with 1–3 loggers per reef zone represented at any particular time. Due to logistical issues, HOBO® temperature logger data are not available for October 2020; no bleaching signs were visually observed on the reef during this sampling point. Based on prior data collected from October 2015 to August 2016, the reef zones in this study had different nutrient concentrations: the fringing reefs on the north shore of Moorea had higher nutrient concentrations, whereas back reef and areas closer to the reef crest generally had lower concentrations [33]. Low nutrient conditions in forereef relative to the back and fringe reef of Moorea have also been documented in [37].

During each sampling point, photographs were taken of each colony and used to visually determine if a colony remained apparently healthy or experienced partial mortality over the course of the study (Fig. 2B). A third category (‘ambiguous’) was used to describe colonies for which health trajectory could not be determined based on the images available (Supplementary Methods). Tissue samples were collected for amplicon sequencing; for each *P. lobata* colony, ~3–6 small fragments (1 cm^2^) of skeleton/tissue were sampled across the colony surface using bone cutters, placed in a sterile Whirl-Pak® (Nasco), and then preserved in DNA/RNA shield (ZymoResearch, Irvine, California, USA). Samples in DNA/RNA shield were kept on ice until returning to shore, at which point they were vortexed for 25 min at full speed with 5 ceramic beads and 1.35 g garnet matrix (MP Biomedicals) and then frozen at −40 °C. An additional sample (~3 cm^2^ fragment) was collected for symbiont density measurements from a subset of these same colonies to compare densities over time and between reef zones. These samples were wrapped in aluminum foil and stored at −40 °C until further processing (see Supplementary Methods).

DNA and RNA were extracted from the coral samples, which included mucus, tissue, and skeleton, using enzyme digestions and a ZymoBIOMICs DNA/RNA Kit (ZymoResearch, Irvine, California, USA, following [29]). Dominant Symbiodiniaceae lineages were identified by amplifying the D1–D2 region of the 28 S large subunit (LSU) nuclear ribosomal RNA gene using the primers LSU1F_illu and LSU1R_illu [38] and processed in RStudio (version 1.1.456) through the DADA2 pipeline (version 1.11.0, [39]) and LULU package, which merges potentially erroneous ASVs based on sequence similarity and co-occurrence patterns ([40], Supplementary Methods). The dinoRNAV major capsid protein (*mcp*) gene was amplified from cDNA (generated from RNA), using a nested PCR protocol with primers from [30]. Sequencing and bioinformatic analyses were conducted following [29] using the program vAMPirus (v1.0.1 [41]). Briefly, after adapter removal, quality filtering, primer removal, read merging, and length filtering, amplicon sequence variants (ASVs) were generated and chimeras removed using VSEARCH with the UNOISE3 algorithm [42, 43]. Parameters and program information for each of these steps can be found in the vAMPirus config file included as a Supplementary File (HoweKerr_etal_2023_vAMPirus.config) and all non-read files used to run the analyses and generate the results can be found at the vAMPirus Zenodo (10.5281/zenodo.7552892). To collapse some of the diversity associated with the high mutation rate of ssRNA viruses [44–46], ASVs were then translated and aligned into unique amino acid types (‘aminotypes’) [29] using VirtualRibosome (v2.0, [47]) and CD-HIT (v.4.8.1, [48]).

### DinoRNAV *mcp* aminotype diversity analyses

All dinoRNAV *mcp* analyses were conducted on samples that produced sequencing libraries containing at least 1,000 reads in R (version 4.1.0). Aminotype richness was calculated with *phyloseq* (estimate_richness function, version 1.36.0) using rarefied data. Linear models were used to assess the influence of reef type and time on aminotype richness using the equation richness~ reef type * time with *stats* (lm function, version 4.1.0). The influence of Symbiodiniaceae density on richness was also assessed, using the equation richness~ density. Normality of residuals was assessed visually with quantile-quantile plots and Shapiro-Wilk tests. Beta diversity analyses were conducted on rlog transformed count data [49] by calculating Bray-Curtis distances with *phyloseq*; within-group distances (or dispersion) were assessed with permutation tests for homogeneity in multivariate dispersion (PERMDISP) using betadisper in *vegan* (version 2.5.7, [50]). Between-group distances (or composition) were assessed with permutational multivariate analysis of variance (PERMANOVA) using adonis in *vegan* and a modified pairwise adonis (following methods in [27]). PERMANOVA were only used to compare groups that met assumptions for homogeneous dispersion between groups (as determined by a non-significant PERMDISP result) since dispersion can impact PERMANOVA inferences [51]. Beta diversity metrics were assessed according to reef type, site, time, colony identity, and colony health (excluding ambiguous gray colonies in Fig. 1B). For some comparisons, samples were categorized according to whether they were collected during ambient conditions on the reef (August 2018 and October 2020 timepoints) versus during the reef bleaching event (March and August 2019 timepoints). Between-group distance comparisons were visualized by plotting the Bray-Curtis distances between samples from one category and the samples of another category. Within-group distances of each sample relative to the centroid of its associated category were graphed and distributions were shown with a boxplot.

A phylogenetic tree was generated to depict relationships between abundant aminotypes (those with at least 10% relative abundance in at least one sample) and related sequences. Sequences included those associated with six coral species with diverse symbionts from the Great Barrier Reef [30], sequences from *Pocillopora* spp. harboring two *Cladocopium* symbionts in French Polynesia [29], and several additional related viral sequences [44, 52–54]. MUSCLEv5 [55] was used to align sequences and Modeltest-ng [56] was used to determine the best model for evolution. A maximum likelihood phylogeny with 500 bootstrap replication steps was generated with Heterocapsa circularisquama RNA virus (HcRNAV, BAE47072.1, HcRNAV109CY, [52]) as an outgroup, using IQ-TREE2 [57]. The tree was visualized using *ggtree* [58]. Branches with <50% bootstrap support were collapsed, and branches with >70% support are marked with a black dot.

## Results

### Temperature and colony health fluctuated across the reef and over time

Overall seawater temperatures (linear model; *R*^2^ = 0.07, *p* < 0.001) and daily temperature ranges (Pairwise Wilcoxon Test, *p* < 0.001 for all) differed significantly among reef zones, with the fringing reef having the highest temperatures (averaging 28.5 ± 1.1 °C and ranging from 21.5 °C to 31.3 °C), and the forereef having the lowest (averaging 27.8 ± 0.9 °C and ranging from 23.5 °C to 30.0 °C; Fig. 2A). March 2019 was the hottest sampling period in every reef zone (Supplementary Table 1), with a mean temperature of 29.4 ± 1.2 °C compared to 27.3 °C ± 0.7 in August 2018 and 27.0 ± 0.5 °C in August 2019 (Pairwise Wilcoxon Test, *p* < 0.01 for all, Fig. 2A). Although this severe island-wide bleaching event was experienced by all reef organisms, bleaching signs within the stony corals were primarily observed in *Acropora* and *Pocillopora* [36, 59] and were evident from March to August 2019 [60]. *Porites lobata* is generally bleaching-resistant [34], and clear visual signs of bleaching were not observed in any focal colonies of this study at any sampling period. Symbiont densities also did not vary by sampling period (Supplementary Fig. 2).

Samples from the 54 colonies were successfully collected at every time point, except for four colonies on the fringe reef, which were not located in October 2020 (Fringe 0: POR578 & 590; Fringe 1: POR75 & 259). All focal coral colonies remained alive over the course of the >3-year sampling period, although 50% (27/54) of colonies exhibited partial mortality (blue colonies in Fig. 1B). Two colonies on the forereef (POR2 and 5) experienced near complete mortality by October 2020, with only small fragments of live tissue remaining and considerable overgrowth of the colony by macroalgae. Colony health varied with reef type (*χ*^2^ = 10.51, *p* < 0.01) and site (*χ*^2^ = 18.37, *p* = 0.01). More forereef corals (78%, 14/18) experienced partial mortality than fringe reef corals (28%, 5/18, *χ*^2^ = 10.49, *p* < 0.01, blue colonies in Fig. 1B).

### *Porites lobata-Cladocopium* C15 colonies contain distinctive dinoRNAV *mcp* aminotypes

In total, 212 samples were collected for amplicon sequencing from the 54 focal colonies over the course of the study. Amplicon sequencing of the Symbiodiniaceae LSU gene indicated that all colonies were dominated by *Cladocopium* C15; *Gerakladium* ASVs were detected in 11% (6/54) of colonies and comprised less than 1% of the total Symbiodiniaceae community in each of these colonies (Supplementary Fig. 3, see Supplementary Results for additional details).

**Fig. 3:**
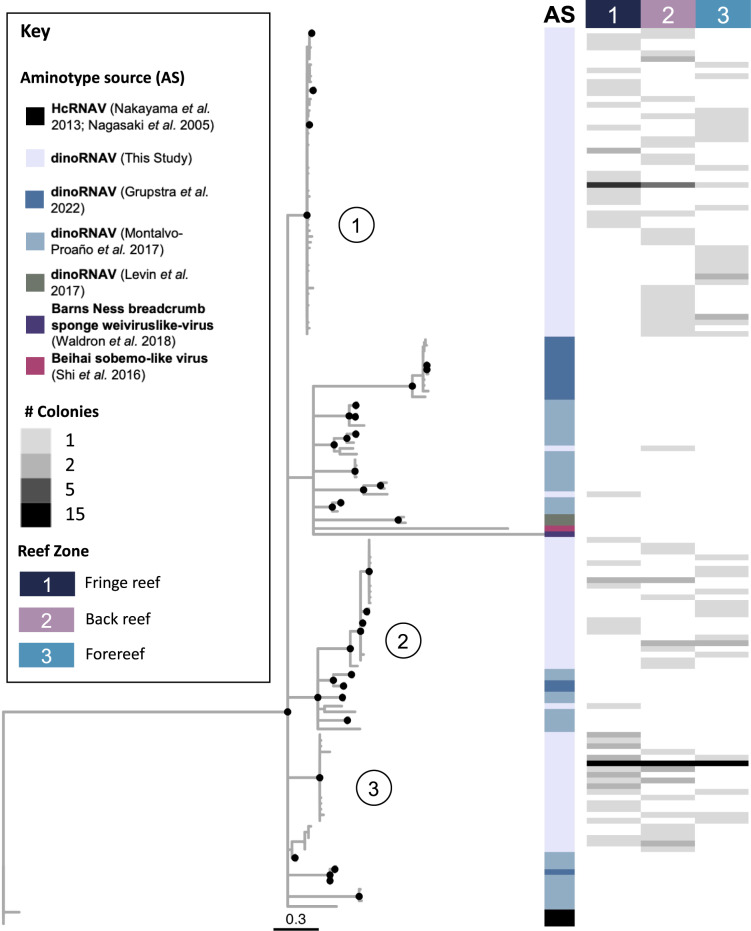
Maximum likelihood tree of dinoRNAV major capsid protein (*mcp*) unique amino acid sequences (‘aminotypes’) from *Porites lobata-Cladocopium* C15 holobionts in this study as well as previously reported dinoRNAV *mcp* sequences and related viral sequences. A maximum likelihood phylogeny with 500 bootstrap iterations was generated using dinoRNAV *mcp* aminotype sequences generated from *Porites lobata-Cladocopium* C15 holobionts from coral reefs of Moorea, French Polynesia (South Pacific) in this study, as well as previously reported *mcp* sequences from the Great Barrier Reef [30], Moorea [29], and related viral sequences such as the Beihai sobemo-like virus and the Barns Ness breadcrumb sponge weiviruslike-virus [44, 54]. This tree was outgroup rooted by the longest branch, Heterocapsa circularisquama RNA virus (HcRNAV, [52, 53]). Colors in the vertical bar (Aminotype Source, AS) indicate the origin of a given sequence. Branches with less than 50% bootstrap support were collapsed; branches with bootstrap support >70% are depicted with a black dot. Only aminotypes that were present in >10% relative abundance in at least one sample are included in the phylogeny. Gray tone heat maps indicate the number of colonies from which each aminotype was detected, separated by reef zone (rightmost columns 1–3). Circled numbers reference the three largest clades of dinoRNAV aminotypes from this study.

Amplicon sequencing of the dinoRNAV *mcp* resulted in 63,378,214 raw reads; 21,165,247 paired reads remained after quality filtering, merging, and length filtering with vAMPirus v1.01. Denoising of unique merged reads and translation of subsequently produced ASVs resulted in 778 unique aminotypes. After exclusion of samples with fewer than 1000 reads, read depth ranged from 1,584 to 361,088 per sample (Supplementary Table 3). Thirteen percent (101/778) of the aminotypes resolved were present in at least 10% relative abundance in at least one sample; these aminotypes were used to calculate and visualize phylogenetic relationships among aminotypes both in this study and from other closely related viruses (Fig. 3). The majority of the aminotypes from this study clustered into three groups; two of these groups formed clades that were distinct from all other sequences in the tree (labeled circles 1 and 3 in Fig. 3). The middle group (labeled circle 2 in Fig. 3) clustered near aminotypes from coral colonies in the Great Barrier Reef [30] and from Moorean *Pocillopora* colonies [29]. Aminotypes from each of these groups were found in all reef habitats (Reef Zone columns 1–3 in Fig. 3). Two aminotypes clustered with dinoRNAV sequences from *Pocillopora* [29], the Barns Ness breadcrumb sponge weivirus-like virus [54] and Beihai sobemo-like virus [44], and dinoRNAV sequences isolated from Symbiodiniaceae in culture [20]. HcRNAV outgroup sequences [52, 53] clustered together in their own clade (Fig. 3).

**Fig. 4:**
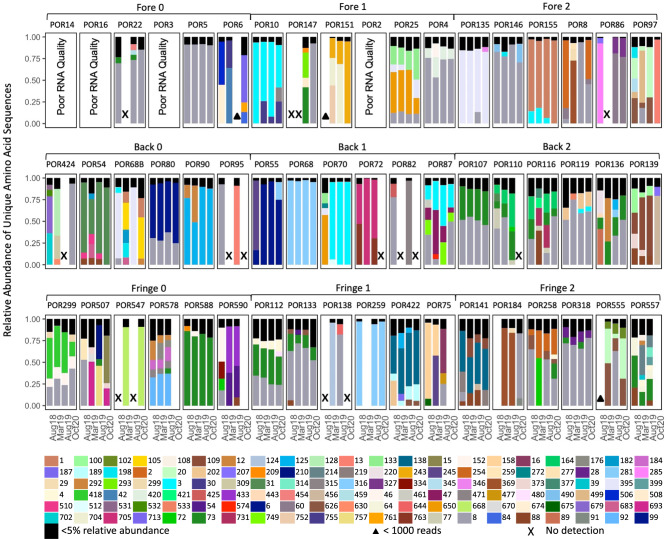
Relative abundance of unique amino acid sequences (‘aminotypes’) detected in *Porites lobata-Cladocopium* C15 holobionts from coral reefs of Moorea, French Polynesia (South Pacific). Each set of four bars represents one colony sampled over time (numerical colony ID above box); colonies are grouped by reef zone (rows) and sites (sequentially ordered across each row). Aminotypes comprising less than 5% relative abundance within a sample are depicted together in black at the top of vertical bars. All other colors represent distinct aminotypes. Triangles indicate positive dinoRNAV detection, but poor sequencing success (<1000 reads); X indicates no detection. Blank space (no symbol) indicates that a sample does not exist for a given time point.

When percent sequence similarity among all 778 aminotypes was compared, aminotypes also clustered into at least three groups, with the largest group encompassing 48% (343/778) of aminotypes (Supplementary Fig. 4). Clustered groups of aminotypes were generally characterized by > 80% sequence similarity, and sequence similarity between groups generally ranged from 40 to 80% (Supplementary Fig. 4). Some aminotypes were highly prevalent. For example, forty-three aminotypes were shared among at least 50% of samples, and four aminotypes (aminotype 626, 650, 73, and 8) were shared between at least 75% of samples. Among these, aminotype 8 was the most common (found in 89% of samples, darkest gray bar in Figs. 3, 4).

### *Porites lobata-Cladocopium* C15 colonies exhibit high dinoRNAV prevalence

The dinoRNAV *mcp* gene was detected in 50 of 54 colonies (93%). Four colonies were excluded from sequencing and analyses due to poor RNA quality (indicated by light gray dots in Fig. 1B), resulting in 196 sequenced samples. Samples with >1000 filtered dinoRNAV *mcp* reads (colored bars in Fig. 4) were considered positive dinoRNAV detections, as were successful PCR amplifications of the *mcp* in-house (black triangles in Fig. 4). Out of the 50 coral colonies from which the dinoRNAV *mcp* was sequenced, dinoRNAV sequences were detected in 91% (179/196) of the samples, and high-quality sequence reads were obtained from 86% (168/196, Fig. 4). Further, 80% (40/50) of colonies exhibited dinoRNAV infection at all timepoints sampled (Fig. 4). The remaining 20% (10/50) of colonies exhibited dinoRNAV infection at some time points, but not all (3/14 colonies on the forereef, 5/18 on the back reef, and 2/18 on the fringing reef; Fig. 4). These non-detection samples were not associated with any particular timepoint. To confirm that quality of cDNA was not the cause of potential false negatives, successful amplification of S-adenosyl *methionine* synthetase, a gene with stable expression in Symbiodiniaceae [61], was used as a positive control. This control gene was successfully amplified in-house from cDNA of all non-detection samples. The threshold for dinoRNAV *mcp* detection (number of infected Symbiodiniaceae cells in a sample required for amplification) is not known, and therefore these samples are referred to as ‘non-detection’ at the colony-level. It is possible that some Symbiodiniaceae cells across the landscape of a coral surface are infected, whereas others are not, potentially affecting detection. Other coral-Symbiodiniaceae holobionts do not always exhibit the high prevalence of dinoRNAV *mcp* detections as reported here. For example, under some conditions, dinoRNAV prevalence in *Acropora hyacinthus*-Symbiodiniaceae holobionts is more heterogeneous and episodic (Supplementary Fig. 5). Taken together, this supports the interpretation of non-detection samples in this study as a biological signal.

DinoRNAV compositions were distinct by colony (PERMANOVA with Bray-Curtis; *F* = 3.46, *R*^2^ = 0.57, *p* < 0.01) with most being dominated by a single aminotype. Seventy-five percent of samples (126/168 colored bars, Fig. 4) had one dominant aminotype (>50% relative abundance, such as aminotype 273 across colony POR68 or aminotype 471 across colony POR318, Fig. 4). Most aminotypes were found in multiple colonies; only 2.2% (17/778) of aminotypes were unique to just one sample.

### Reef zone influenced dinoRNAV *mcp* aminotype richness and composition

Seventy percent of aminotypes were shared across the three reef zones (Fig. 1C), whereas fewer than 4% of aminotypes were unique to one reef zone (Fig. 1D). Aminotypes from all three predominant clades in Fig. 3 were found in all reef habitats (Reef zone columns 1–3 in Fig. 3).

**Fig. 5:**
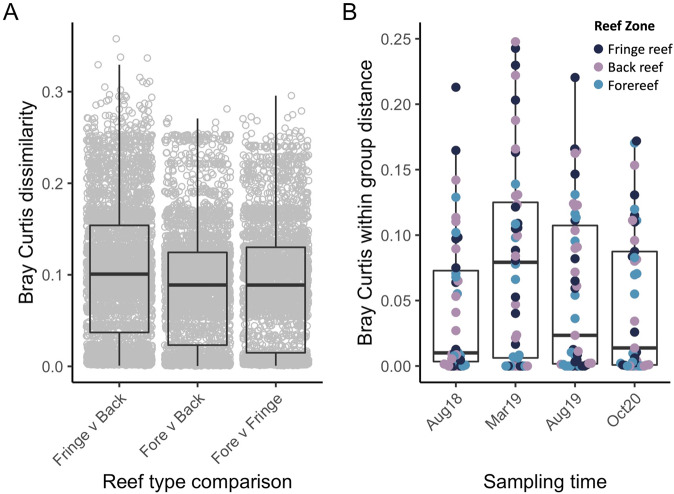
Bray-Curtis beta diversity metrics of dinoRNAV *mcp* aminotypes from *Porites lobata-Cladocopium* C15 colonies varied by reef type and sampling time on the reefs of Moorea, French Polynesia (South Pacific). **A** Between-group distances (composition) varied by reef type, with the fringe and back reef differing the most. The boxplot displays Bray-Curtis distances of dinoRNAV *mcp* aminotypes from samples of each pairwise combination of reef types. Each dot represents the Bray-Curtis distance between a sample from one reef type and a sample of another reef type. **B** Within group distances (dispersion) varied across sampling timepoint. The boxplot displays mean within-group distances of dinoRNAV *mcp* aminotypes for each sample, separated by sampling timepoint. Each dot represents one sample; dots are colored according to reef type.

Aminotype richness differed by reef zone (ANOVA, *F* = 5.93, *p* < 0.01) and was highest in the fringing reef (fringe: 146 ± 62.4; back: 123 ± 67.7; fore: 107 ± 40.6; fringe relative to fore: Pairwise Wilcoxon Test, *p* = 0.06, Fig. 1E). Dispersion (i.e., within group distance) did not vary by reef type (PERMDISP with Bray-Curtis, *F* = 1.63, *p* = 0.19; Supplementary Fig. 6), while aminotype composition (i.e., between-group distance) did vary by reef type (PERMANOVA with Bray-Curtis; *F* = 1.95, *R*^2^ = 0.02 *p* = 0.02, Fig. 5A); pairwise tests showed that fringe reef aminotypes differed significantly from the back reef (*R*^2^ = 0.02, *p* = 0.05), and the forereef trended towards differing from the fringe and the back in composition as well (*R*^2^ = 0.02, *p* = 0.09 for both comparisons, Fig. 5A). When aminotype composition of samples in each reef type was analyzed separately, site significantly influenced composition in the fringe (PERMANOVA with Bray-Curtis; *F* = 1.89, *R*^2^ = 0.61, *p* = 0.04) and back (*F* = 2.22, *R*^2^ = 0.07, *p* = 0.02) reefs, but not in the forereef (*F* = 0.76, *R*^2^ = 0.03, *p* = 0.59). Aminotype richness did not correlate with Symbiodiniaceae density (linear model; *F* = 0.04, *R*^2^ < 0.01, *p* = 0.85, Supplementary Fig. 7).

### Dispersion of dinoRNAV *mcp* aminotypes was highest during the reef-wide bleaching event, and colonies that exhibited partial mortality differed in dinoRNAV *mcp* aminotype composition

**Fig. 6:**
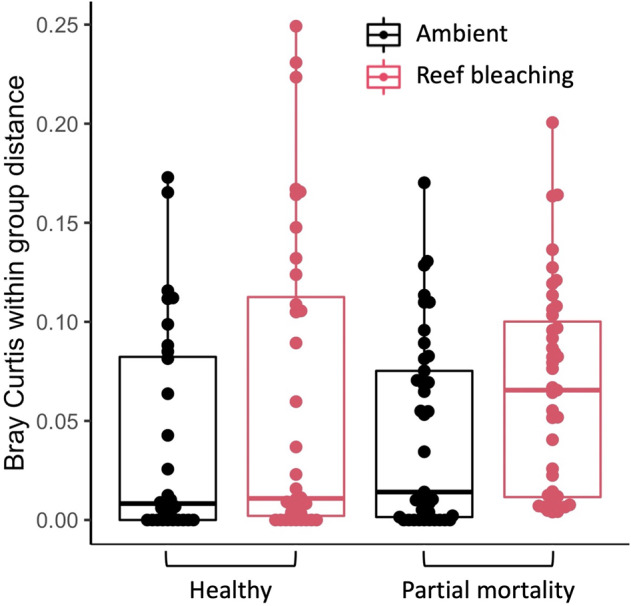
Dispersion (within group distances) of dinoRNAV *mcp* aminotypes from *Porites lobata-Cladocopium* C15 colonies varied by thermal stress timepoint and colony health trajectory on the reefs of Moorea, French Polynesia (South Pacific). Dispersion was overall higher in samples collected during reef bleaching timepoints. August 2018 and October 2020 samples were grouped as ambient temperature timepoints; March and August 2019 were grouped as “reef bleaching” related timepoints since they fell at the beginning and end of the bleaching event in Moorea. Colonies were grouped according to their health trajectory over the sampling duration (colonies that did not exhibit partial mortality- ‘healthy’ vs. colonies that did- ‘partial mortality’). Colonies with ambiguous health trajectories were excluded.

With all reef types considered together, aminotype dispersion varied across sampling timepoint (Fig. 5B). Dispersion was highest during the bleaching event in March 2019 (Fig. 5B) and trended towards significant differences (PERMDISP with Bray-Curtis, *F* = 2.43, *p* = 0.06). When samples from colonies with ambiguous health trajectories were excluded and timepoints were grouped according to their proximity to the reef bleaching event, dispersion was significantly higher during reef bleaching timepoints compared to ambient temperature timepoints (PERMDISP with Bray-Curtis, *F* = 5.14, *p* = 0.03, red boxplots in Fig. 6). When colonies that exhibited partial mortality versus healthy colonies were analyzed separately, the pattern of elevated dispersion during reef bleaching conditions was strongest (and trended towards significance) in colonies with partial mortality (PERMDISP with Bray-Curtis, *F* = 3.50, *p* = 0.07 vs. healthy colonies: *F* = 2.0, *p* = 0.17, Fig. 6). Overall, while dispersion did not vary according to colony health trajectory (PERMDISP with Bray-Curtis, *F* = 0.15, *p* = 0.71), colony health trajectory significantly correlated with composition of dinoRNAV aminotypes (PERMANOVA with Bray-Curtis; *F* = 1.98, *R*^2^ = 0.013, *p* = 0.03). There was not an interaction between health trajectory and ambient versus bleaching timepoints (PERMANOVA with Bray-Curtis; *F* = 0.51, *R*^2^ = 0.01, *p* = 0.99).

## Discussion

Most shallow-water coral holobionts live in conditions that are close to the upper limit of their thermal tolerance [62, 63]. As ocean temperatures intermittently surpass these thresholds, it is critical to understand the infection dynamics of dinoRNAVs and other viruses that potentially impact coral-Symbiodiniaceae interactions. Similarly, densely populated nearshore areas (e.g., fringing reefs) are typically more nutrient-rich than offshore (e.g., forereef) habitats, which can lead to increased abundances of microorganisms, including viruses, in nearshore habitats [64]. Although we have abundant evidence from ‘omics-based approaches that corals harbor a diverse virosphere [10, 11, 13, 65–67], the distribution of these viruses in space and time as well as their infection dynamics are not well resolved. Here, we characterized the diversity and infection prevalence of dinoRNAVs in dominant reef-building *Porites lobata* colonies across multiple years and reef environments. Over 90% of the sampled colonies had detectable dinoRNAV infections, whose composition and diversity differed among reef zones, indicating that environmental conditions were deterministic in viral-host dynamics. DinoRNAV aminotype variability increased during the reef-wide thermal stress event, suggesting an increase in viral productivity, and this pattern was strongest in the colonies that exhibited partial mortality during the study. Such lineage-specific inferences advance coral reef virology by identifying host-virus interactions that may drive ecosystem impacts.

### DinoRNAV diversity is highest in the fringing reef and aminotype composition varies by reef zone

*Porites lobata* colonies are found in the fringe, back and forereef zones of Moorea and maintain a highly specific association with *Cladocopium* C15 Symbiodiniaceae in all zones (Supplementary Fig. 3). We leveraged this consistent host-symbiont system to test multi-year dinoRNAV dynamics across different environmental conditions. The composition of viral aminotypes was influenced by reef zone; within the back and fringe reef, there was spatial variation at the site level as well (e.g., aminotype 8 in Back 1 vs. Back 2 in Fig. 4). The forereef, which had the lowest richness and no spatial (site) variation, had more homogenous environmental conditions ([33], Supplementary Fig. 1B); statistical power was also more limited for this reef zone due to poor RNA quality in some samples (Fig. 4 top row). In contrast, the fringe and back reef had more variable conditions, resulting in finer-scale variation in viral aminotype assemblages. The differences in viral diversity and composition according to reef zone suggest that environmental conditions across a reefscape exert distinctive selective pressures [68, 69] and dinoRNAVs may vary in their productivity across a reef.

High intra-colony dinoRNAV richness in the fringing reef colonies (Fig. 1E) may be indicative of increased viral productivity associated with one or more elevated abiotic conditions in this environment. The fringing reef has the highest and most variable temperatures (Fig. 2, Supplementary Fig. 1B), and high temperature was previously attributed to inducing shifts to more productive modes of viral infection [29, 70]. The fringing reefs on the north shore of Moorea also have the highest nutrient concentrations due to coastal runoff [33], and therefore higher abundances of fleshy macroalgae [71]. This could contribute to higher microbial respiration, which has been attributed to viral productivity in other systems [72]. Nutrients may also increase Symbiodiniaceae growth rates [73–75] triggering more productive dinoRNAV infections. However, we did not document elevated Symbiodinaceae densities in the fringing reef colonies (Supplementary Fig. 2). Elevated levels of one or more of these conditions on the fringing reef, a result of both the physical parameters of the reef and proximity to human activity, appear conducive to viral infections.

### Dinoflagellate-infecting RNA viruses are diverse, common, and persistent in *Porites lobata-Cladocopium* C15 colonies

*Porites lobata* are stress-resistant coral holobionts that will likely become an even more dominant player on Indo-Pacific reefs as climate change drives losses in more susceptible taxa [76]. DinoRNAV infection prevalence is high within *P. lobata-Cladocopium* C15 colonies in Moorea; dinoRNAV *mcp* aminotypes were detected from almost every colony sampled, and at all timepoints. Since the only colonies from which dinoRNAVs were not detected were four colonies with poor RNA quality, the infection prevalence of dinoRNAVs at the colony level may even exceed the 93% (50/54) reported here (see Supplementary Fig. 5 for evidence supporting that high dinoRNAV infection prevalence in *P. lobata-Cladocopium* C15 in this study is a biological signal and not a methodological artifact). Symbiodiniaceae are vertically transmitted in *Porites* eggs, and this mode of transmission may contribute to high colony-level infection prevalence. The dinoRNAV detections in this study are most parsimoniously interpreted as representative of active infections, rather than endogenized viral elements (EVEs) in the Symbiodiniaceae genome. This is because (1) a genome-wide exploration of dinoRNAVs detected zero *mcp* EVEs in a *Cladocopium* C15 genome [77], and (2) *mcp* genes were successfully amplified from unfractionated coral cDNA but were not amplifiable from DNA purified from the same samples in parallel (*n* = 8, data not shown). The 778 detected aminotypes encompass at least three distinct groups of dinoRNAVs, based on phylogenetic and sequence similarity (Fig. 3, Supplementary Fig. 4). These groups are potentially indicative of ‘quasispecies’, a term often used to describe RNA viral diversity since RNA polymerase (RdRp) has high error rates during replication [78–82]. Deciphering the dynamics of these active viral infections is integral to understanding the persistence of robust *Porites* colonies in climate-stressed reefs.

### DinoRNAVs are more productive during thermal stress and in colonies with declining health

Temporal patterns in aminotype diversity metrics revealed a signature of the reef bleaching event in *P. lobata-Cladocopium* C15 colonies. When timepoints were grouped according to their proximity to the reef bleaching event (timepoints of ambient temperature before and after 2019 versus the 2019 timepoints that fell during the bleaching event), dispersion of aminotypes was significantly higher during the reef bleaching timepoints. This pattern was primarily driven by colonies exhibiting partial mortality (Fig. 6), suggesting a connection between colony health, thermal stress, and dinoRNAV productivity. Heat may trigger increased viral productivity (as evident in non-coral systems [70, 83]), driving declines in Symbiodiniaceae health and indirectly impacting coral colony health. Alternatively, as Symbiodiniaceae and/or coral colonies become physiologically stressed [84], viral productivity may increase as a secondary infection that results in additional physiological stress. In addition to examining host immune system responses (of both coral and Symbiodiniaceae), quantitative approaches such as real-time PCR and detection of dinoRNAVs though Fluorescence in situ Hybridization [85] may be helpful in determining viral load and resolving functional roles of dinoRNAV infections at the resolution of the Symbiodiniaceae population, since coral holobionts harbor millions of Symbiodiniaceae cells per square centimeter of coral tissue [86]. Overall, our results show that in a bleaching-resistant coral-holobiont system, dinoRNAVs respond to in situ heat stress and have potentially nuanced, non-lethal health impacts on coral colonies.

Direct impacts of higher viral productivity on Symbiodiniaceae—the hosts of dinoRNAVs—may differ from the net impact of this viral lineage on the coral holobiont [1], particularly since Symbiodiniaceae and coral can interact on a continuum from parasitism to mutualism [87, 88]. For example, Symbiodiniaceae can become selfish under elevated nutrient conditions, sequestering nutrients from the coral host [75]; if dinoRNAV infections then cause Symbiodiniaceae population declines, the coral may ultimately benefit in this circumstance. The interplay of this tripartite association will likely shift as conditions on coral reefs continue to rapidly change.

### Defining the functional roles of viruses that infect Symbiodiniaceae

The role of viruses as pathogens has been emphasized in plant virology, especially in agriculturally relevant crops [3, 89]. Similarly, the role of viral lysis in coral bleaching and disease [11, 14, 29, 66] has been a primary investigative focus in economically and ecologically valuable coral reef environments. However, there is increasing recognition that plant viruses in natural systems can spread widely without causing severe damage to their hosts [2, 3, 79, 82], and can even increase plant fitness in some circumstances [4]. Especially in systems like *Porites lobata-Cladocopium* C15 colonies that are long-lived and do not frequently bleach, it is also important to consider the diversity of potential outcomes in a coral-dinoflagellate-virus symbiosis [1].

There may be contexts in which Symbiodiniaceae viruses can benefit their algal hosts or the coral holobiont at large [90, 91]. In the plant holobiont, *Dichanthelium lanuginosum*, thermal tolerance is elevated when the fungal partners of a grass are infected with a virus that alters its gene expression [92]. Viral genes encoding for photosynthetic proteins have been documented in corals and similarly have been thought to compensate for photosynthetic damage to Symbiodiniaceae during heat stress [10, 20]. Selective infection and lysis of certain taxa can also modulate a microbial symbiont community in ways that benefit the holobiont, such as has been found with bacteriophage [93–96]. Coral holobionts can sometimes harbor multiple Symbiodiniaceae taxa, and these taxa may have competitive interactions [24, 25, 97, 98]; Symbiodiniaceae viruses could hypothetically alter the outcomes of those interactions if infections are host specific. Finally, elevated Symbiodiniaceae densities can be detrimental to coral hosts (discussed in [75, 99, 100]), and symbiont viruses could help maintain symbiont populations. Deciphering these possible roles for Symbiodiniaceae-infecting viruses will require further lineage-specific investigations of infection prevalence within individual host cells, and quantification of the physiological impacts these infections have on hosts under different environmental contexts.

## Conclusions

By quantifying dinoRNAV prevalence in individual colonies across years in a bleaching-resistant coral holobiont, this study shows that dinoRNAVs are a persistent component of a multi-partite symbiosis. Environmental conditions throughout the reef shaped the composition of viral *mcp* aminotypes, as evidenced by compositional differences according to reef type and site. Patterns in viral diversity (dispersion and richness of aminotypes) suggest that temperature can increase dinoRNAV productivity with potential connections to host and colony health. Infection prevalence and fitness impacts at the cellular level in Symbiodiniaceae constitute an important next step to deciphering the functional roles of dinoRNAVs. Taken together, our findings provide the first characterization of coral-holobiont viruses at the reef scale and demonstrate that infection patterns of dinoRNAVs appear to be driven, at least in part, by environmental fluctuations that will intensify under global climate change.

## Supplementary Information

ISMEJCOMMS-22-00210A-T-s01

ISMEJCOMMS-22-00210A-T-s02

ISMEJCOMMS-22-00210A-T-s03

## Data Availability

All *mcp* gene amplicon libraries and Symbiodiniaceae LSU gene amplicon libraries are available at the Sequence Read Archive under accession numbers PRJNA928208 and PRJNA930706, respectively.
