# Supplementary material for: Viruses of a key coral symbiont exhibit temperature-driven productivity across a reefscape
Source: ISME Commun. 2023 Apr 3;3:27. doi: 10.1038/s43705-023-00227-7 (PMC10068613; doi:10.1038/s43705-023-00227-7)
Supplement: Supplementary file 1 — ISMEJCOMMS-22-00210A-T-s01 [file 43705_2023_227_MOESM1_ESM.docx]

**Viruses of a key coral symbiont exhibit temperature-driven productivity across a reefscape**

Howe-Kerr *et al.* Supplementary Materials

**Supplementary Methods**

***Colony image analysis***

Photographs of each colony were taken at each sampling point and used in visual assessments to determine if a colony remained apparently healthy or experienced partial mortality over the course of the study; a third category (‘ambiguous’) was used to describe colonies for which health trajectory could not be determined based on the images available. Colonies were considered to have experienced partial mortality if there was a clear loss of live tissue surface area between August 2018 and October 2020; colonies were considered to have remained apparently healthy if there was an increase in live tissue in images taken over the course of the assessed period or if there was no clear change in live tissue surface area. Final health determination was based on assessments by three separate observers, who assessed the images separately and blindly without knowing what reef zone or site a set of images was collected from. Differences in coral health among reef types and sites were assessed with Chi Squared tests.

***Symbiodiniaceae cell density measurements***

Symbiodiniaceae densities were calculated from a subset of colonies to compare densities over time and reef zones. These samples were wrapped in foil and stored at -40 °C until further processing. Frozen coral fragments were thawed, airbrushed with 0.2 μm filtered seawater (FSW) to remove coral tissue from the skeleton, and then the separated tissue was homogenized using a Fisherbrand 150 handheld homogenizer (Waltham, Massachusetts) for ten seconds at full speed. The total homogenized tissue volume was recorded for standardization of Symbiodiniaceae cell densities to fragment surface area. Symbiodiniaceae cell densities were quantified by preserving a 250 μl aliquot of the same homogenized coral tissue as above in 10% formaldehyde in FSW. Cells were counted using a Neubauer hemocytometer at 20X magnification (n = 2 technical replicates/sample) and concentrations were normalized to fragment surface area. Fragment surface area was calculated by uploading ~150 images of each coral fragment taken at 10° and 60° angles into Agisoft Metashape (St. Petersburg, Russia), generating a 3D model, and measuring the resultant tissue surface area.

***Sequencing, bioinformatic processing, and analysis of Symbiodiniaceae LSU rRNA gene***

To identify dominant Symbiodiniaceae lineages, the D1–D2 region of the 28S large subunit (LSU) nuclear ribosomal RNA gene was amplified from coral holobiont DNA. This marker was selected because, in a recent consensus statement by Symbiodiniaceae researchers, it was recommended to consider the use of some other marker genes (including the LSU 28S) that, although technically multicopy, behave as though they are single copy, since the majority of copies present are a single sequence [1]. At the Oregon State University Center for Qualitative Life Sciences (CQLS), PCR reactions were conducted using the primers LSU1F_illu and LSU1R_illu [2] with attached Miseq Adapters; cycles were as follows: 95°C for 3 min, and then 15 cycles of 95°C for 30 sec, 56°C for 30 sec, and 72°C for 30 sec, and finally, 72°C for 4 min. PCR clean-up was completed using Agencourt AMPure XP Magnetic Beads. PCR reactions to incorporate Illumina indexing primers were conducted as follows: 95°C for 3 min, and then 20 cycles of 95°C for 30 sec, 56°C for 30 sec, and 72°C for 30 sec, and finally 72°C for 4 min. The resulting PCR product was purified with Agencourt AMPure XP Magnetic Beads, quantified via qPCR using the KAPA library quantification kit (Roche Sequencing Solutions, Pleasanton, CA), pooled in equal molar amounts, and sequenced on the Illumina MiSeq platform with PE300 chemistry.

Amplicons of the LSU gene were processed in RStudio (version 1.1.456) through the DADA2 pipeline (version 1.11.0), which generates unique sequences at single-nucleotide resolution (amplicon sequence variants or ‘ASVs’) [3]. Forward and reverse paired reads were truncated at 210 and 160 base pairs, respectively, based on quality plots. Reads with a total expected error of one or more, or contained Ns, were discarded. ASVs were inferred from unique reads, paired reads were merged, ASVs that did not match a target length of 286 ± 5 were removed, and chimeras were detected and removed. This DADA2 curated table of ASVs were then further collapsed using the LULU package [4], which merges potentially erroneous ASVs based on sequence similarity and co-occurrence patterns (default parameters of 84% similarity, 90% co-occurrence used here; see github.com/CorreaLab/POR_dinoRNAV_MVP for pre- and post-LULU ASVs). Taxonomy was assigned to curated ASVs based on BLAST (blastn) searches to NCBI’s nr/nt database, and ASVs that had best matches to non-Symbiodiniaceae were discarded.

**Supplementary Results**

**Experimental Design and Temperature Results**

**
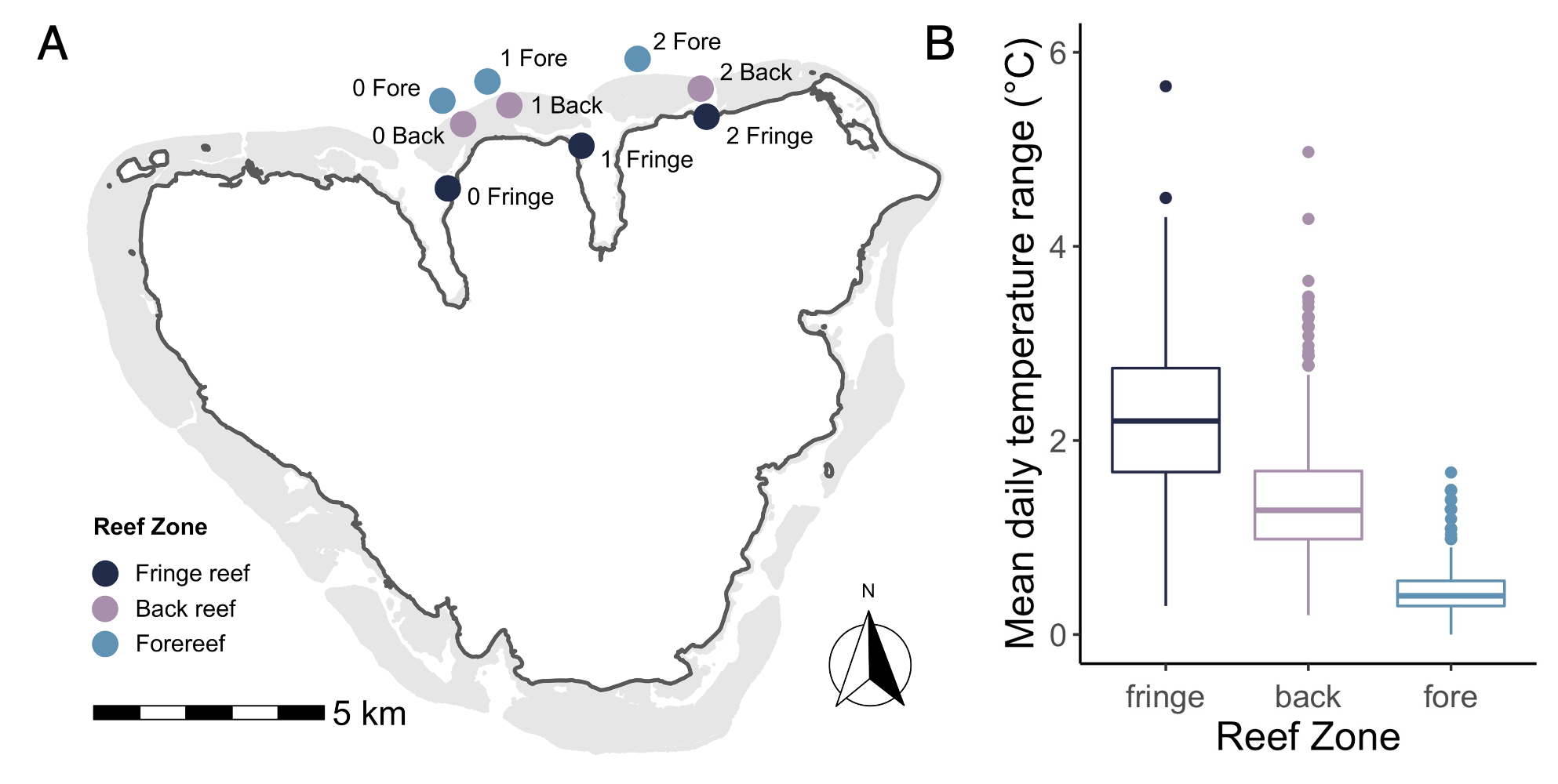
**

**Supplementary Figure 1.** Study site locations and temperatures from coral reefs of Moorea’s north shore (French Polynesia, South Pacific). (A) The nine reef sites where *Porites lobata* colonies (n = 6 per site) were tagged and sampled from August 2018 to October 2020. (B) Mean daily temperature range for each of the reef types, averaged from HOBO® temperature logger data collected from the reef zones from August 1, 2018- September 1, 2019. Color denotes reef type in both panels.

***Seawater temperatures varied by reef zone and were highest during the March 2019 sampling point***

The fore reef had the lowest temperatures, averaging 27.8 ℃ ± 0.9 and ranging from 23.5 ℃ to 30.0 ℃. Back reef temperatures averaged 28.4 ℃ ± 1.1 and ranged from 24.3 ℃ to 30.9 ℃. The fringe reef had the highest temperatures, averaging 28.5 ℃ ± 1.1 and varying the most over time with a range of average daily temperatures from 21.5 ℃ to 31.3 ℃. Daily average reef temperatures across the north shore were highest during the March 2019 sampling time point (Supp. Table 1). Temperatures were significantly higher in March 2019 for every timepoint (Pairwise Wilcoxon Tests with Bonferroni correction, p <0.005 for each reef zone, August 2018 vs March 2019).

**Supplementary Table 1:** Mean seawater temperatures for the month of each sampling period, separated by reef zone, for coral reefs of Moorea’s north shore (French Polynesia, South Pacific). Mean temperatures for the full month were calculated from HOBO® temperature logger data; at any given month, data from 1-3 temperature loggers per reef zone are represented.

| **Reef Zone** | **Sampling Month** | **Mean Temperature (°C) ± SD** |
| --- | --- | --- |
| Fringe | August 2018 | 27.6 ± 0.9 |
|  | March 2019 | 30.3 ± 1.7 |
|  | August 2019 | NA |
| Back | August 2018 | 27.3 ± 0.8 |
|  | March 2019 | 29.8 ± 0.7 |
|  | August 2019 | 27.5 ± 0.9 |
| Fore | August 2018 | 27.2 ± 0.3 |
|  | March 2019 | 28.5 ± 1.1 |
|  | August 2019 | 26.9 ± 0.2 |

**Symbiodiniaceae Results:**

***Colonies were dominated by consistent densities of Symbiodiniaceae***

A subset of colonies from each reef zone were sampled for Symbiodiniaceae density at each time point (n = 13 during August 2018; n = 8 during March 2019; n = 11 during August 2019), generating a total of 32 samples. Symbiodiniaceae densities averaged 9.0x10^5^ ± 7.9x10^5^ (fringe), 7.8x10^5^ ± 4.7x10^5^ (back), and 4.3x10^5^ ± 2.1x10^5^ (fore) mean cells/ cm^2^ ± sd (Supp. Fig. 3). Densities did not vary significantly by time or reef zone (Pairwise Wilcoxon Test with Bonferroni correction, p > 0.1 for all comparisons).


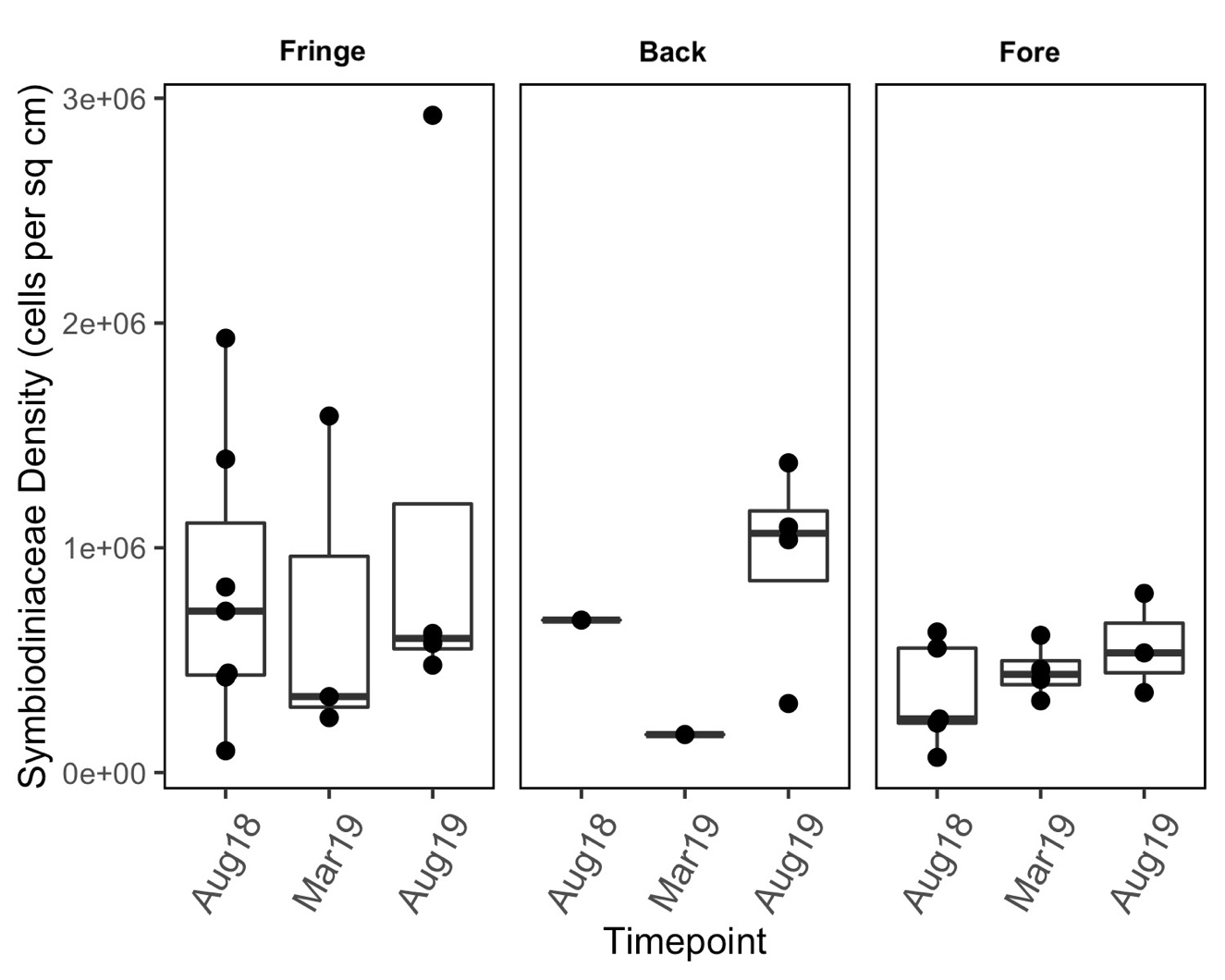


**Supplementary Figure 2.** Symbiodiniaceae densities from a subset of *Porites lobata* colonies sampled in the fringing, back and forereef of Moorea’s north shore (French Polynesia, South Pacific) in August 2018, March 2019, and August 2019. Densities did not vary significantly by time (Pairwise Wilcoxon Test with Bonferroni correction, Aug18 vs Mar19 p = 1, Mar19 vs Aug19 p = 0.19; Aug18 vs Aug19 p = 0.91) or reef zone (Pairwise Wilcoxon Test with Bonferroni correction, Fringe vs Back p = 1, Back vs Fore p = 0.54; Fringe vs Fore p = 0.29).

***Colonies were dominated by* Cladocopium *Symbiodiniaceae***

In total, 212 samples were collected for amplicon sequencing from the 54 focal colonies over the course of the study. Amplicon sequencing of the Symbiodiniaceae LSU gene produced 13,832,837 raw reads; after quality filtering and processing through DADA2, including merging of paired end reads, 11,929,901 paired reads remained and were used to infer ASVs. Following target length trimming and chimera removal, 335 ASVs were ultimately identified. Processing the resultant ASVs through LULU resulted in 17 final ASVs. Based on BLAST (blastn) searches to NCBI’s nr/nt database, 13 ASVs did not show strong similarity to Symbiodiniaceae (non-target amplification) and were therefore excluded, leaving 8-33,848 reads per sample. Samples containing fewer than 300 reads (n = 47 of 212 samples) were discarded. All remaining reads belonged to four ASVs; two ASVs had best BLAST hits to *Cladocopium* *C15* and two had best hits to *Gerakladium* (Supp. Table 2). Each of the 54 focal colonies had at least one sampled time point that produced a sequence library with over 300 reads. Overall, LSU sequencing indicated that all colonies were dominated by *Cladocopium C15* (Supp. Fig. 2); *Gerakladium* ASVs were detected in 11% (n = 6 of 54) of colonies and comprised less than 1% of the total Symbiodiniaceae community in each of these colonies (represented as green or yellow dots above vertical bars in Supp. Fig. 2).

**
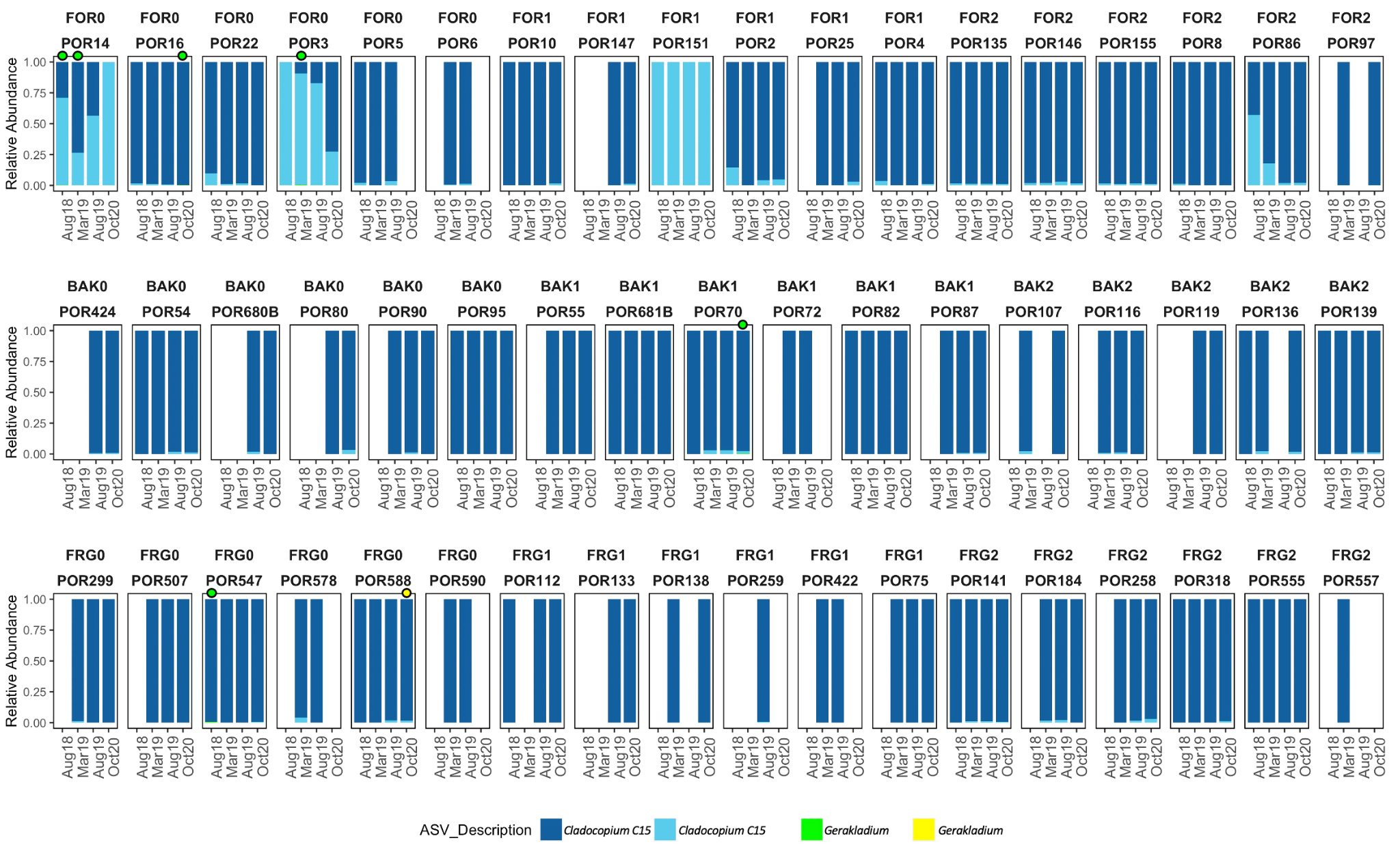
**

**Supplementary Figure 3.** Relative abundance of Symbiodiniaceae amplicon sequence variants from the D1–D2 region of the 28S large subunit (LSU) nuclear ribosomal RNA gene sampled from *Porites lobata* colonies on the fore, back and fringe reefs of Moorea’s north shore (French Polynesia, South Pacific) in August 2018, March 2019, August 2019 and October 2020. Dots above sample bars indicate that *Gerakladium* was detected in <0.1% relative abundance. Samples are arranged by reef type (rows) and grouped by colony ID and site.

**Supplementary Table 2:** Curated Symbiodiniaceae amplicon sequence variants from the D1–D2 region of the 28S large subunit (LSU) nuclear ribosomal RNA gene sampled from *Porites lobata* colonies on reefs of Moorea’s north shore (French Polynesia, South Pacific). ‘ASV sequences’ below are the end product of ASVs produced with the DADA2 [3] pipeline and subsequently curated with the LULU package [4]. The ‘Accession #’ column displays the top hit and associated statistics (query coverage, E value and % identity) for each ASV sequence (note that for the topmost ASV sequence, the Accession # indicates the sixth-best hit, which was annotated as *Cladocopium C15*; the first five hits were to unannotated *Porites*-associated Symbiodinaceae). ‘Lineage ID’ indicates the lineage each ASV sequence is interpreted to belong to for the purposes of this study.

| **ASV sequence** | **Accession #** | **Lineage ID** |
| --- | --- | --- |
| ATAGGATTCCCTCAGTAATGGCGAATGAACAGGGATTGGCTCAGGCTGGAAACTGAAGCTTCGGTTTCGGGATGTAGCCTGCAGGCATAGTGCTATCGGCGGCTCGAGCGTAAGCCTCTTGGAACAGAGCGTGTGCCCGGGTGAGAATCCTGTGTTTCGCTTGATGTCCGCTGTCCACAGCACTTGTTCTCAGAGTCACGCTCCTCGGAATTGGAGCGTAAATAAGGTGGTAAATTTCATCTAAAGCTAAATACGGGCTCGAGACCGATAGTGAACAAGTACCA | JN558044.1  Query Cover: 100%  E value: 4e-143  % Identity: 99.65% | *Cladocopium C15* |
| ATAGGATTCCCTCAGTAATGGCGAATGAACAGGGATTGGCTCAGGCTGGAAACTGAAGCTTCGGTTTCGGGATGTAGCCTGCAGGCATAGTGCTATCGGCGGCTCGAGCGTAAGCCTCTTGGAATAGAGCGTGTGCCCGGGTGAGAATCCTGTGTTTCGCTTGATGTCCGCTGTCCACAGCACTTGTTCTCAGAGTCACGCTCCTCGGAATTGGAGCGTAAATAAGGTGGTAAATTTCATCTAAAGCTAAATACGGGCTCGAGACCGATAGTGAACAAGTACCA | JN558044.1  Query Cover: 100%  E value: 8e-145  % Identity: 100% | *Cladocopium C15* |
| ACAGGATTCCCTTAGTAATGGCGAACGAACAGGGAAACGCTCAGGCTTAAAACCAAGGCTCTGGTTTTGGGTTGTAGCCTGCAGATATTGCGCTATCGGTGGCTCGAGCGTAAGCCTCTTGGAATAGAGCGTAAACTTGGGTGAGAATCCTTTGTTTCGCTCGGTGTCCGCTGTGTACGGCGCATGTTCTCAGAGTCACGTTCCTCGGACTTGGAGCGTAAATTAGGTGGTAGATTTCATCTAAAGCTAAATAAAGGCCCAAGACCGATAGCGAACAAGTACCA | MF322789.1  Query Cover: 100%  E value: 8e-145  % Identity: 100% | *Gerakladium* sp. |
| ACAGGATTCCCTTAGTAATGGCGAACGAACAGGGAAACGCTCAGGCTTAAAACCAAGGCTCTGGTTTTGGGTTGTAGCCTGCAGATATTGCGCTATCGGTGGCTCGAGCGGAAGCCTCTTGGAATAGAGAGTAAACTTGGGTGAGAATCCTTTGTTTCGCTCGGTGTCCGCTGTGTACGGCGCATGTTCTCAGAGTCACGTTCCTCGGACTTGGAGCGTAAATTAGGTGGTAGATTTCATCTAAAGCTAAATAAAGGCCCAAGACCGATAGCGAACAAGTACCA | MF322789.1  Query Cover: 100%  E value: 2e-141  % Identity: 99.30% | *Gerakladium* sp. |


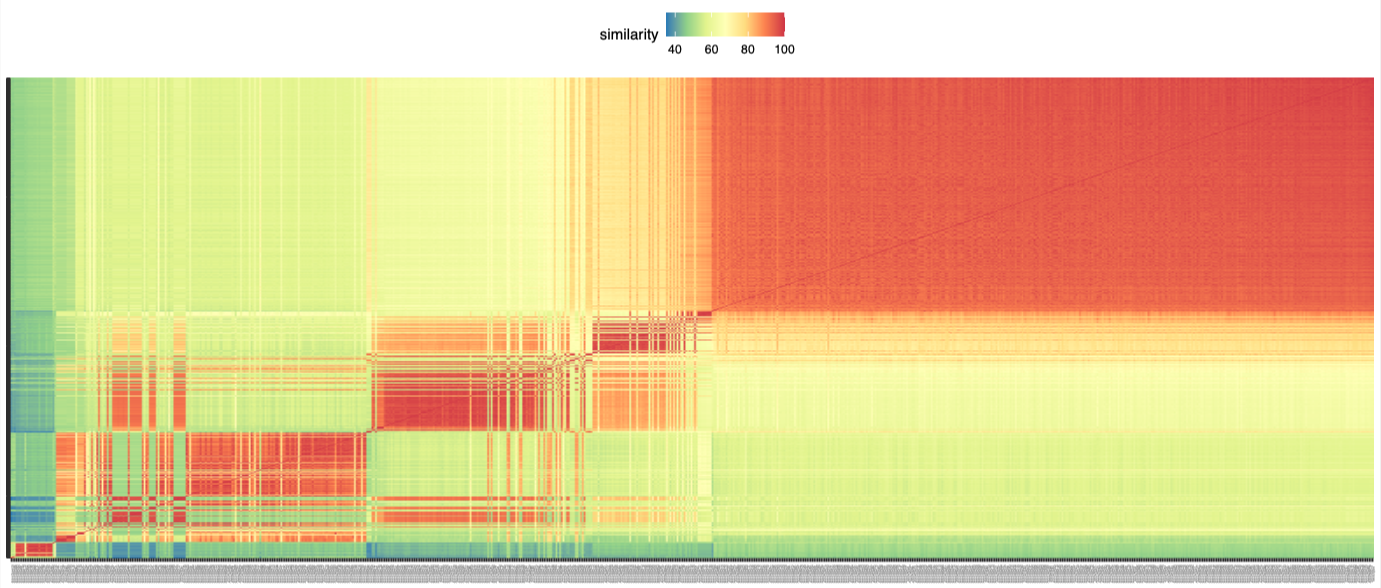


**Supplementary Figure 4.** Similarity matrix of all unique amino acid sequences (‘aminotypes’) detected in *Porites lobata-Cladocopium C15* holobionts from coral reefs of Moorea, French Polynesia (South Pacific). Axes are ordered by aminotypes, and color denotes percent aminotype similarity.


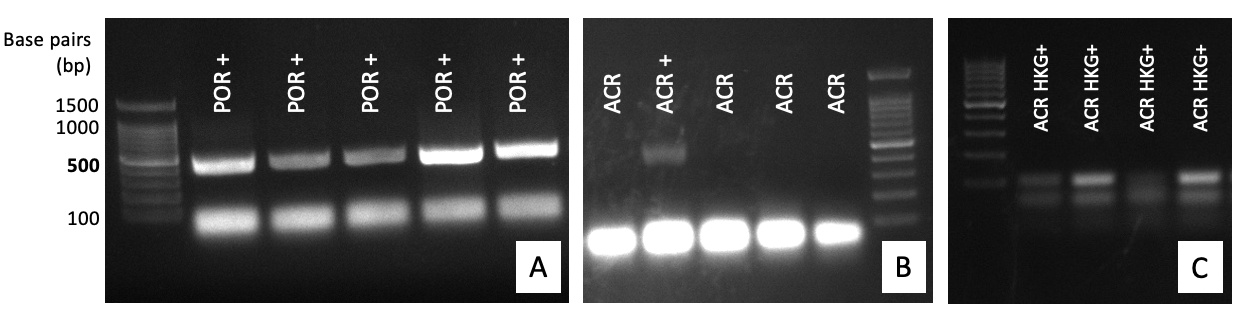


**Supplementary Figure 5.** Agarose gels showing representative amplification of the dinoRNAV major capsid protein (*mcp*) gene from two coral species (*Porites lobata* and *Acropora hyacinthus*), as well as a stably expressed housekeeping gene *(A. hyacinthus*). A ladder ranging from 100-1500 is visible on either the left or right edge of all gel images. POR indicates a *P. lobata* sample (this study) and ACR indicates an *A. hyacinthus* sample (unpublished data). (A-B) The dinoRNAV *mcp* gene was amplified from cDNA (generated from RNA), using a nested PCR protocol with primers from Montalvo-Proaño *et al.* 2017 [5] that produced a ~500 bp amplicon. DinoRNAVs were detected in five of the five *P. lobata* samples shown, as indicated by the five lanes labeled ‘POR +’ in (A), but in only one of the five *A. hyacinthus* samples shown, as indicated by the lane labeled ‘ACR +’ in (B). (C) Example gel image of *A. hyacinthus* samples from which the dinoRNAV *mcp* did not amplify, but a housekeeping gene (HKG) with stable expression in Symbiodiniaceae, S-adenosyl methionine synthetase, did amplify using primers from Rosic *et al.* 2011 [6] (~100 bp bands labeled ‘ACR HKG +’). Positive amplifications of the HKG confirm that cDNA quality was sufficient to support amplification of the dinoRNAV *mcp* gene (if present) in these samples. In other words, positive amplification of the HKG from a sample was used to rule out a false negative for dinoRNAV infection, when dinoRNAV *mcp* amplicons were not observed.

**
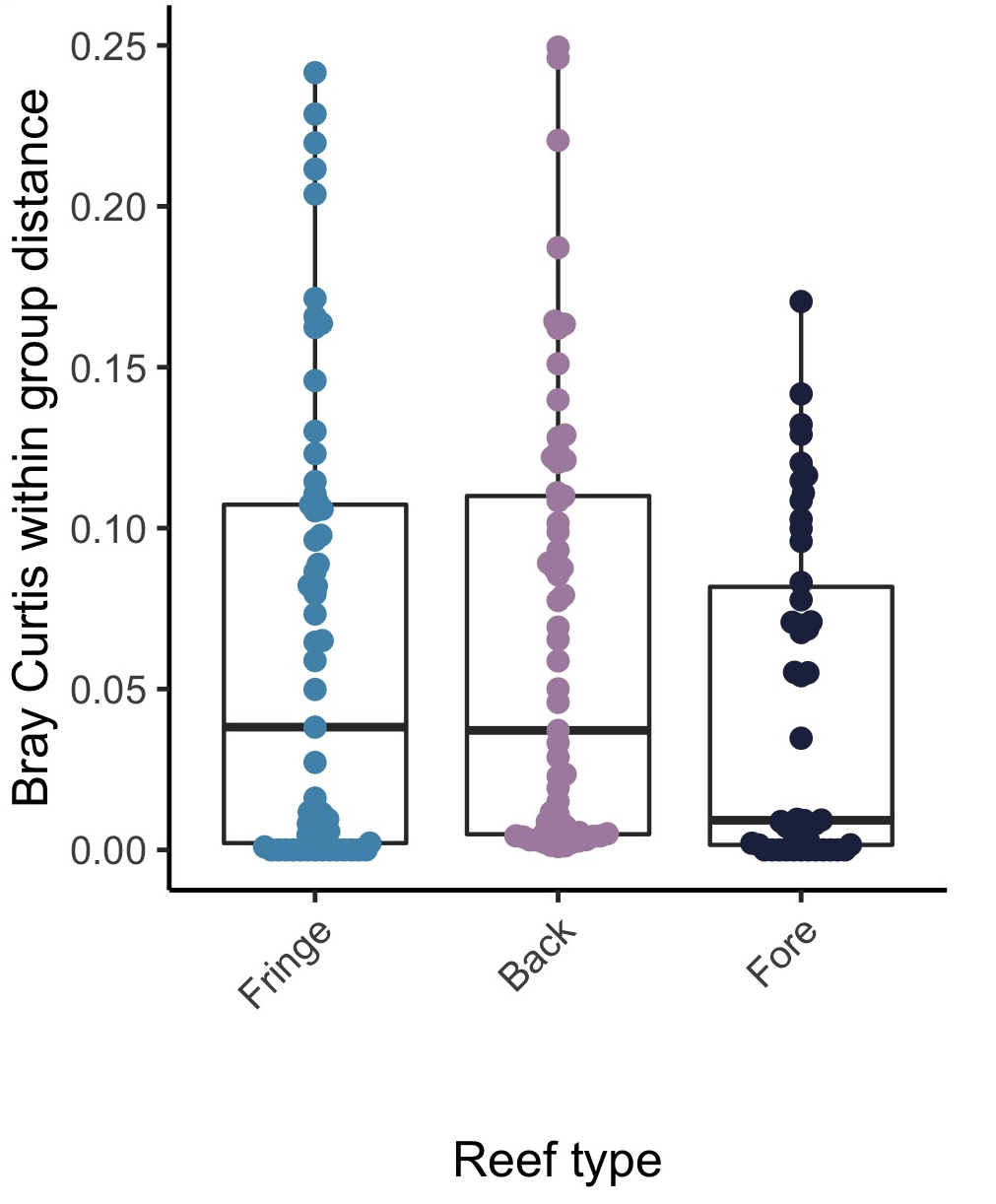
**

**Supplementary Figure 6.** Dispersion, or within-group distances, of dinoRNAV *mcp* aminotypes from *Porites lobata-Cladocopium C15* colonies on the reefs of Moorea, French Polynesia (South Pacific) did not vary significantly by reef zone (PERMDISP with Bray-Curtis, F = 1.63, p = 0.19). Each dot overlaying the boxplot represents one sample.

**
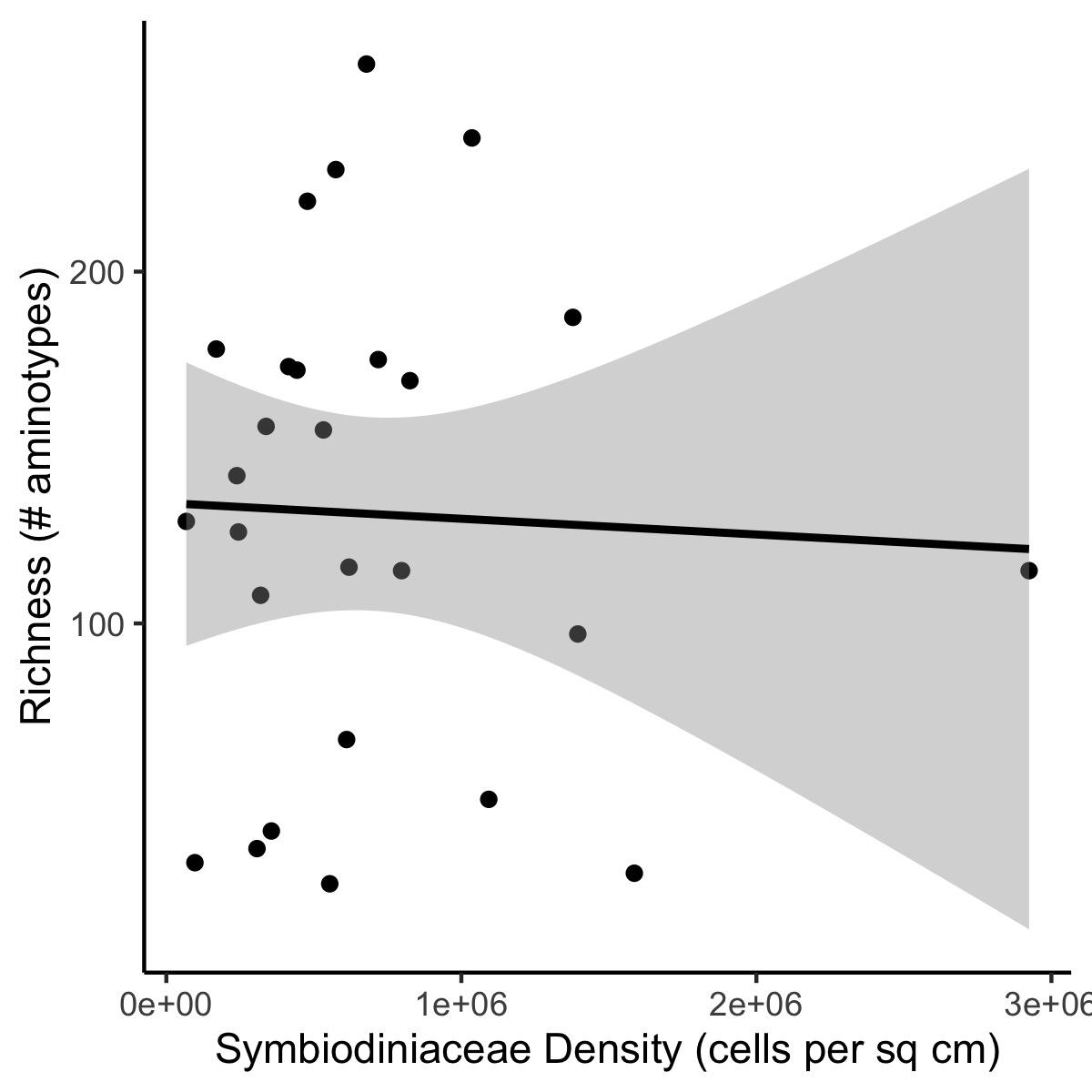
**

**Supplementary Figure 7.** Richness of unique amino acid sequences (‘aminotypes’) detected in *Porites lobata-Cladocopium C15* holobionts from coral reefs of Moorea, French Polynesia (South Pacific) was not associated with holobiont Symbiodiniaceae densities, based on a linear model (R^2^ = 0.001, F = 0.04, p = 0.85).

**References**

1. Davies, S, Gamache MH, Howe-Kerr LI, Kriefall NG, Baker AC, Banaszak AT., et al. Building Consensus around the Assessment and Interpretation of Symbiodiniaceae Diversity. Preprints 2022; 2022060284.
2. Pochon X, Wecker P, Stat M, Berteaux-Lecellier V, Lecellier G. Towards an in-depth characterization of Symbiodiniaceae in tropical giant clams via metabarcoding of pooled multi-gene amplicons. *PeerJ* 2019; **7**: e6898.
3. Callahan BJ, McMurdie PJ, Rosen MJ, Han AW, Johnson AJA, Holmes SP. DADA2: High-resolution sample inference from Illumina amplicon data. *Nature Methods* 2016; **13**: 581–583.
4. Frøslev TG, Kjøller R, Bruun HH, Ejrnæs R, Brunbjerg AK, Pietroni C, et al. Algorithm for post-clustering curation of DNA amplicon data yields reliable biodiversity estimates. *Nat Comms 2017 8:1* 2017; **8**: 1–11.
5. Montalvo-Proaño J, Buerger P, Weynberg KD, van Oppen MJ. A PCR-based assay targeting the major capsid protein gene of a dinorna-like ssRNA virus that infects coral photosymbionts. *Front Microbiol* 2017; **8**.
6. Rosic NN, Pernice M, Rodriguez-Lanetty M, Hoegh-Guldberg O. Validation of Housekeeping Genes for Gene Expression Studies in *Symbiodinium* Exposed to Thermal and Light Stress. *Mar Biotechnol* 2011; **13**: 355–365.
